# A Multidisciplinary Investigation into the Talent Development Processes at an English Football Academy: A Machine Learning Approach

**DOI:** 10.3390/sports10100159

**Published:** 2022-10-19

**Authors:** Adam L. Kelly, Craig A. Williams, Rob Cook, Sergio Lorenzo Jiménez Sáiz, Mark R. Wilson

**Affiliations:** 1 Research Centre for Life and Sport Sciences (CLaSS), Faculty of Health, Education and Life Sciences, Birmingham City University, Birmingham B15 3TN, West Midlands, UK; 2 Children’s Health and Exercise, Research Centre and Sport and Health Sciences, College of Life & Environmental Sciences, University of Exeter, Exeter EX1 2LU, Devon, UK; 3 Centre for Sport Studies, Universidad Rey Juan Carlos, Fuenlabrada, del Molino, 5, 28942 Madrid, Spain

**Keywords:** talent identification, expertise, psychological characteristics, physical characteristics, technical and tactical, elite youth soccer

## Abstract

The talent development processes in youth football are both complex and multidimensional. The purpose of this two-fold study was to apply a multidisciplinary, machine learning approach to examine: (a) the developmental characteristics of under-9 to under-16 academy players (*n* = 98; Study 1), and (b) the characteristics of selected and deselected under-18 academy players (*n* = 18; Study 2). A combined total of 53 factors cumulated from eight data collection methods across two seasons were analysed. A cross-validated Lasso regression was implemented, using the glmnet package in R, to analyse the factors that contributed to: (a) player review ratings (Study 1), and (b) achieving a professional contract (Study 2). Results showed non-zero coefficients for improvement in subjective performance in 15 out of the 53 analysed features, with key findings revealing advanced percentage of predicted adult height (0.196), greater lob pass (0.160) and average dribble completion percentage (0.124), more total match-play hours (0.145), and an older relative age (BQ1 vs. BQ2: −0.133; BQ1 vs. BQ4: −0.060) were the most important features that contributed towards player review ratings. Moreover, PCDEQ Factor 3 and an ability to organise and engage in quality practice (PCDEQ Factor 4) were important contributing factors towards achieving a professional contract. Overall, it appears the key factors associated with positive developmental outcomes are not always technical and tactical in nature, where coaches often have their expertise. Indeed, the relative importance of these factors is likely to change over time, and with age, although psychological attributes appear to be key to reaching potential across the academy journey. The methodological techniques used here also serve as an impetus for researchers to adopt a machine learning approach when analysing multidimensional databases.

## 1. Introduction

It is widely acknowledged that the talent development processes in youth football are both complex and multidimensional [1]. Although various factors have been identified to influence the talent development processes in youth football, only a few multidisciplinary studies exist. As an example, Huijgen and colleagues [2] applied a battery of objective field tests and questionnaires within the four domains of technical, tactical, physiological, and psychological characteristics to players aged 16 to 18 years. It was revealed that selected players outperformed their deselected counterparts, whereby performance in the technical skill of dribbling, the tactical characteristics of positioning and deciding, and the physiological attribute of sprinting correctly classified 69% of talented players. Moreover, Forsman and colleagues [3] examined multiple factors of youth football players at aged 15 years that eventually contributed to successful football performance at aged 19 years. Performance at aged 19 years was associated with technical (i.e., passing), tactical (i.e., centering), physiological (i.e., agility), and psychological (i.e., motivation) attributes that were displayed at aged 15 years. In addition, Zuber and colleagues [4] observed holistic patterns as an instrument for predicting the performance in promising young football players over a three-year period. They revealed that highly skilled players scored above average on all technical, physiological, and psychological factors. Collectively, this research reinforces the importance of providing a multidimensional research methodology in youth football when exploring the talent development processes.

The multidisciplinary nature of the talent development process is also reflected in several theoretical (e.g., Personal Assets Framework [5,6]) and practical (e.g., Locking Wheel Nut Model [7]) frameworks. One practically based model that is particularly relevant to the talent development processes in youth football is The Football Association’s Four Corner Model (FCM) [8]. The FCM is often adopted in professional football clubs and organisations in England (amongst other countries), which advocates the assessment and development of players according to: (a) *technical/tactical*, (b) *physical*, (c) *psychological*, and (d) *social* attributes. Towlson and colleagues [9] applied the FCM to their qualitative methodology whilst examining the perceived importance that practitioners placed on the four sub-components during player selection in academy football. It was discovered that the psychological sub-component was rated significantly higher than the other three sub-components. Likewise, Kelly and colleagues [10] adopted the FCM in a quantitative analysis of factors differentiating those who ‘play-up’ an age-group compared to those who do not based on age phase (i.e., Foundation Development Phase [FDP]: under-9 to under-11; Youth Development Phase [YDP]: under-12 to under-16). Technical/tactical and social characteristics appeared to differentiate those who play-up compared to those who do not at ages 8 to 11 years, whereas there were measures representing all four sub-components from the FCM for those aged 11 to 16 years. Since the FCM is a tool that is perceived to be relevant and useful for football coaches and practitioners [11,12], it provides a salient framework for understanding the factors associated with talent development and thus may facilitate important knowledge translation.

With talent development being inherently multifactorial, explorative studies must employ analysis techniques that can handle multiple competing, and possible correlated, features. Traditional regression techniques inherently struggle to estimate model coefficients when the number of independent variables (IVs) is comparable to the number of observations, though the emerging family of feature selection algorithms from the machine learning discipline offer possible solutions. In the case of regression, feature selection is often achieved by including a penalization term during the model fit, such as the Lasso first proposed by Tibshirani [13], creating a scenario whereby the optimal model is that which explains the most of the data with the fewest parameters. These penalized regression routines present themselves as a potential tool for rapidly summarising observational retrospective data, as well as generating new insights and testable hypotheses. The advantage of such approaches is that they can effectively process large amounts of data for key features in a cost effective and timely manner. Within the remit of talent development, the machine learning approaches here do not aim to answer the deep questions of what leads to optimal performance, but instead seek to demonstrate a method to leverage some of the quantities of available data to generate new hypotheses and insights [14].

Although the use of machine learning as a statistical analysis method in sport science research is very much in its infancy, there is an increasing amount of literature that has applied such methods, including competition outcome predictions [15], human movement [16], practice history [17], and injury risk [18]. From a talent development perspective, preliminary studies in cricket have used non-linear machine learning (pattern recognition) techniques to examine various factors that contribute to ‘super-elite’ status [19,20]. As an example, Jones and colleagues [19] showed how a subset of 18 features (from 658 collected) differentiated ‘super-elite’ (i.e., high-profile international) and ‘elite’ (domestic professional) senior batsmen with excellent classification accuracy (96%). Moreover, Musa and colleagues [21] classified and predicted ‘high-potential’ archers from a set of variables trained on a variation of k-NN algorithms and logistic regression. Weighted k-NN outperformed all the tested models with reasonably good accuracy (83%) for the prediction of ‘high-potential’ (i.e., top of group) and ‘low-potential’ (i.e., bottom of group) developmental level (aged 13 to 20 years) archers. Most recently, Owen and colleagues [22] used a Bayesian machine learning approach to identify the physiological and psychosocial models that predict selection to a regional age-grade rugby union team. They showed their physiological models correctly classified 67.55% of all players, whereas their psychosocial models correctly classified 62.26% of all players.

It is also important to consider the intra- and inter-contextual factors when designing and evaluating talent development processes, since predictive features (i.e., technical/tactical, physical, psychological, social) in youth football can differ compared to other sports (e.g., cricket, archery, rugby union) and within football-specific environments (e.g., soccer, beach soccer, sepak takraw [23]), respectively. Thus, this current study aims to add to the growing body of literature that has applied machine learning techniques in sport to better understand talent development processes in youth football. The purpose of this two-fold study was to use machine learning algorithms to: (a) explore the multidimensional developmental characteristics of under-9 to under-16 football academy players based on coaches review ratings, and (b) examine the multidimensional characteristics that differentiated selected and deselected under-18 football academy players based on achieving a professional contract.

## 2. Exploring the Developmental Characteristics of under-9 to under-16 Football Academy Players

Professional football academies and governing bodies aim to foster player development pathways towards expertise through adopting evidence-based philosophies (see the Elite Player Performance Plan via The Premier League [24]). In England, young players join an academy on schoolboy terms between the ages of 8 and 16 years (i.e., part-time attendance). At aged 16 years, those players who show continued progress are selected to undertake a two year, full-time youth training scheme known as an academy scholarship. Upon completion of their scholarship, players either sign a professional contract or are released. These developmental stages have been divided into three phases to capture the possible age-specific requirements: (a) FDP (under-9 to under-11), (b) YDP (under-12 to under-16), and (c) Professional Development Phase (PDP; under-17 to under-21). In the pursuit of developing male players towards their respective senior team, professional clubs and organisations continue to invest a significant monetary outlay towards human (e.g., coaches, specialist support staff) and physical (e.g., facilities, specialist equipment) resources [25]. In order to better understand the talent development processes in youth football, it is important to identify factors that may influence the progression of schoolboy players (i.e., FDP and YDP). By doing so, it will also help inform key stakeholders (e.g., coaches, practitioners, policy makers) to create evidence-based policies that will offer each individual the most suitable opportunity to achieve a scholarship and professional contract (i.e., PDP).

The purpose of this study was to examine a range of factors based on the FCM (i.e., technical/tactical, physical, psychological, social) that may have contributed to under-9 to under-16 academy football players’ review ratings across two seasons using a machine learning approach.

### 2.1. Methods

#### 2.1.1. Sample

Ninety-eight male participants were recruited from under-9 to under-16 age groups. All the participants were from the same tier four English professional football club and their category three academy. The average weekly training and match-play time was 9–10.5 training hours/week with one match-play hour/week for the FDP players, and 10–14.5 training hours/week with one match-play hour/week for the YDP players. Goalkeepers were not included in this study due to their contrasting position-specific requirements [26]. Parental consent and player assent were collected prior to the study commencing. The study was approved by the Ethics Committee of Sport and Health Sciences at the University of Exeter.

#### 2.1.2. Measures and Procedures

**Figure 1:**
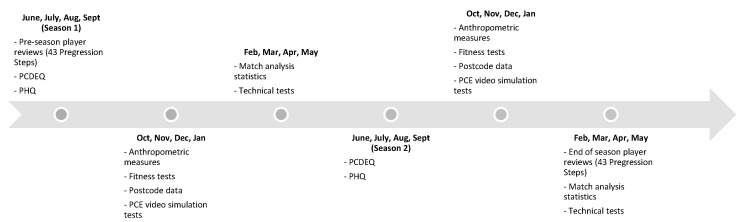
Timeline of the data collection.

The dataset comprised of eight data collection methods that were collected twice during two football seasons (2014–2015 and 2015–2016) to test year-on-year developmental outcomes (see Figure 1 for a timeline of the data collection). These measures were then allocated into the four sub-components in-line with the FCM: (1) *Technical/Tactical*; (a) technical tests [27,28], (b) match analysis statistics [28], and (c) perceptual-cognitive expertise (PCE) video simulation tests [29]. (2) *Physical*; (a) anthropometric measures, and (b) fitness tests [29]. (3) *Psychological*; (a) the Psychological Characteristics for Developing Excellence Questionnaire (PCDEQ) [30,31,32]. And, (4) *Social*; (a) Participation History Questionnaire (PHQ) [33], and (b) postcode data [30].

A combined total of 53 characteristics were cumulated from the eight methods [34]: (1) *Four football-specific technical tests*; (a) ball juggling, (b) slalom dribble, (c) shooting accuracy, and (d) lob pass. (2) *Eight match analysis statistics from across the entire season*; (a) reliability in possession, (b) pass completion, (c) tackle completion, (d) block completion, (e) loose balls retrieved, (f) dribble completion, (g) total touches, and (h) goals scored. (3) *Three PCE video simulation tests*; (a) ‘pre’ execution occlusion, (b) ‘during’ execution occlusion, and (c) ‘post’ execution occlusion. (4) *Eight anthropometric measures*; (a) height, (b) body mass, (c) body mass index, (d) body fat percentage, (e) estimated adult height, (f) percentage of estimated adult height attained, (g) maturity status, and (h) birth quartile. (5) *Eight fitness tests*; (a) 0–10 m sprint test, (b) 0–30 m sprint test, (c) 10–30 m sprint test, (d) L-agility left test, (e) L-agility right test, (f) L-agility test combined, (g) countermovement jump height, and (h) countermovement jump flight time. (6) *Six factors from the 59-item PCDEQ*; (a) Factor 1 (support for long-term success), (b) Factor 2 (imagery use during practice and competition), (c) Factor 3 (coping with performance and developmental pressures), (d) Factor 4 (ability to organise and engage in quality), (e) Factor 5 (evaluating performances and working on weaknesses), and (f) Factor 6 (support from others to compete to my potential). (7) *Ten items from the PHQ*; (a) age started playing football, (b) age started playing academy football, (c) total match-play hours, (d) total coach-led practice hours, (e) total peer-led play hours, (f) total individual practice hours, (g) total football hours, (h) total sports played, (i) total multisport hours, and (j) total football and multisport hours. Finally, (8) *six measures from postcode data*; (a) home area code, (b) home financial risk, (c) home social classification, (d) school area code, (e) school financial risk, and (f) school social classification. The procedures for each of these methods have been outlined in our previously published work [27,28,29,30,31,32,33,34], which have been added below (Section 2.1.2) for the convenience of the reader.

##### Four Football-Specific Technical Tests

Four football-specific technical tests were used to measure technical ability: (a) ball juggling, (b) slalom dribble, (c) shooting accuracy, and (d) lob pass. First, the slalom dribble test required the player to control the ball through nine cones (2 m apart) from the start to the end line and return. The timings were recorded using timing gates (Brower TC Timing System, Draper, Utah, USA), with each player completing two trials and the quicker of the two recorded for analysis. Second, the lob pass test required the player to kick the football from a distance of 20 m into a target area divided into three concentric circles (3 m, 6 m, and 9.15 m in diameter). Each kick was scored by the circle in which the ball initially landed (3, 2, and 1 point, respectively). Ten attempts (five with each foot) were executed with a maximum of 30 points available. Third, the shooting accuracy test required the player to kick the ball at a 16 m wide goal target from a shooting distance of 20 m and central to the goal. The goal was divided into five parallel zones, whereby the centre was, 2 m wide (3 points), with two areas 3 m on each side of the centre (2 points), and two areas 4 m wide at each extreme (1 point). Ten attempts (five with each foot) were executed with a maximum of 30 points available. Fourth, the ball juggling test required the player to keep a football off the ground with the total number of touches recorded. Two trials were completed, with a maximum of 100 touches per attempt permitted, allowing a maximum number of 200 touches. Each player completed these tests in an indoor sports hall with a hard-wood floor, with generic training kit being worn. In addition, age group-specific balls were used for the tests in-line with the Football Association regulations, with size three for under-9, size four for under-10 to under-13, and size five for under-14 to under-16 [27,28].

##### Eight Match Analysis Statistics from across the Entire Season

Video footage examined each player during competitive match-play as they performed each skill behaviour. An average score of each skill behaviour was computed from across an entire football season, including: (a) reliability in possession percentage, (b) pass completion percentage, (c) number of tackles, (d) number of blocks, (e) number of loose balls retrieved, (f) successful dribble completion, (g) total touches, and (h) goals scored. As a standard pro-forma of match analysis statistics within each academy varies based on its philosophy, this current study applied the academy’s existing protocol for its data collection. The specialist software Gamebreaker^©^ was used to perform participant analysis for each game and trained, club-appointed Performance Analysts (who were not part of the research team and were blind to the grouping of the study participants) adopted technical expert definitions to code behaviours (*n* = 10). Twenty matches (25% of the data) of the matches that were included in the current study were used to calculate the Performance Analysts’ reliability (15-day test–retest analysis). One match per team was randomly selected to carry out the intra- and inter-reliability analysis. An intra-class correlation coefficient test was executed to analyse the reliability levels (poor, <0.50; moderate, 0.50 to 0.75; good, 0.76 to 0.90; excellent, 0.91 to 1.00). Results showed the intra-observer reliability ranged from 0.76 to 1.00 and the inter-observer reliability ranged from 0.71 to 1.00 [28].

##### Three Perceptual-Cognitive Expertise Video Simulation Tests

Film-based simulation tests were applied to examine the players’ decision-making skill. Action sequences were selected from live football match footage of academy players aged 18 to 19 years engaging in a competitive game, filmed from an elevated angle above and behind the goal. Following general build-up play of five to ten seconds in duration, the clips unexpectedly occlude immediately prior to a critical decision moment. At this point, an occlusion display appears that shows the pitch lines (i.e., boundaries, eighteen yard box, and half way line) and the location of the ball on a white screen. This screen was frozen for 7-s whereby the participant had to select their answer on the response sheet before the next clip automatically begins. Forty-five clips were created for three different phases that were used for analysis: (a) ‘pre’, (b) ‘at’, and (c) ‘post’ execution. Thus, 135 clips were viewed by the players in total. ‘Pre’ clips are considered more difficult as the occlusion happens 0.5 s prior to the action that is executed, whereas the ‘at’ clips occlude during the moment the action is executed, as opposed to the ‘post’ clips that are considered the easiest as they are occluded after the execution with a duration 0.5 s longer. Consequently, clips are viewed in this order, with a response sheet completed separately and collected before the next batch of clips begin, to prevent players changing their answer when they see the longer clips. The 45 film-based simulations are distributed into three decision-making skills, including ‘select action’, ‘select direction’, and ‘select pass recipient’, thus creating 15 clips for each [29].

##### Eight Anthropometric Measures

The physiological measures included: (a) height, (b) body mass, (c) body mass index (BMI), and (d) body fat percentage. Height measures were recorded to the nearest 0.1 cm (Seca 213 Leicester Height Measure). Body mass measures were recorded to the nearest 0.1 kg (Tanita BF-350 Body Composition Monitor). Body mass index was calculated through dividing weight (kg) by height (m) and dividing that number by height (kg/m²). Body fat percentage was also estimated (Tanita BF-350 Body Composition Monitor). Players completed these procedures bare footed with their training shorts and t-shirt on. Moreover, the Khamis-Roche method was used to analyse: (a) predicted adult height, (b) percentage of predicted adult height attained, and (c) PHV status. The Khamis-Roche method is based on a mathematical calculation using the child’s gender, current height and body mass, and the height of both parents. The formula applied to predicted height in inches is: =((age factor) * (age in years)) + ((height factor) * (height in inches)) + ((body mass factor) * (body mass in pounds)) + ((parental height factor) * (parental height in inches)) + (beta coefficient) [35]. The participants predicted adult height then identifies the percentage of predicted adult height attained. Additionally, the growth curve attained from this data identifies the participants PHV status: (a) pre-, (b) circa-, and (c) post-PHV. Lastly, birth quartile was measured by dividing the twelve months of the year into four quarters, conforming to the strategy applied to distribute chronological age groups. Due to the start of the section year beginning in September in England, this is recognised as ‘month 1’ while August is ‘month 12’.

##### Eight Fitness Tests

Fitness tests were conducted with the participants to measure specific physical parameters, including acceleration, sprint, agility, and jump abilities. These tests were executed by the first author and have been proved valid and reliable measures for talent development research in youth football. Players were already familiarised with these testing procedures since they were already part of the academy fitness testing protocol. The 0–30 m sprint test started 1 m behind the first set of timing gates (Brower TC Timing System, Draper, UT, USA). Participants sprinted until passing the final set of timing gates. Timings for 0–10 m, 0–30 m, and 10–30 m were taken to observe acceleration and sprint speed, respectively. The L-agility test required the participants to start 1 m behind the first set of timing gates (Brower TC Timing System, Draper, UT, USA), then run forwards 5 m around the tall centre cone, run 5 m to the left hand cones and place one foot between the two marker cones, and then turn and follow the same path back to the start. In the second trial, players performed the same test, but this instance running 5 m to the cones on the right-hand side. Timings were recorded for the right, left, and combined. During the CMJ test (Just Jump system, Probotics Inc. 8602 Esslinger CT, Huntsville, AL, USA), players were instructed on the importance of using a countermovement and the need to take-off and land with straight legs, with the jump height (cm) and time (s) recorded for analysis. Three trials were completed for each test with the best result taken for investigation. Players conducted these fitness tests in a sports hall, whilst players completed a familiarity session prior to the data collection to counteract any earning effects.

##### Six Factors from the 59-Item Psychological Characteristics for Developing Excellence Questionnaire

The 59-item PCDEQ was used to assess psychological characteristics across six dimensions. Each of the questionnaire’s items is placed on a six-point Likert scale with a similarity response method from ‘1’ (very unlike me) to ‘6’ (very like me). This ensured participants were not allowed to remain neutral and therefore encouraged them to think more carefully about whether they agree or disagree with the statement leading to greater accuracy. Additionally, a mixture of positively and negatively worded items is included to minimise the danger of acquiescent bias. The PCDEQ is designed for youth athletes, thus offers user-friendly language that is applicable to this cohort (see MacNamara and Collins [31] for the psychometric properties of the PCDEQ). The participants completed the PCDEQ in a classroom setting. They were allocated 45-min to complete it and the researcher was available to help answer any questions if the participants were unsure.

##### Ten Items from the Participation History Questionnaire

The PHQ is a retrospective recall questionnaire, which is used to elicit information regarding the activities in which players have engaged in during their development. The test–retest reliability and the concurrent validity of the PHQ have been previously established by Ford and colleagues [33]. The PHQ contains three sections including milestones within football, engagement within football activities, and engagement in other sport activities. Initially, the football-specific milestones include both: (a) the age at which the player first engaged in football, and (b) the age they began participation in a professional football academy. The second section of the PHQ is designed to elicit information from four football-specific activities: (a) match-play, (b) coach-led practice, (c) individual practice, and (d) peer-led play. The hours per week and months per year in each of these football activities, as well as the accumulation of time spent engaged in all of these activities, were recorded in the PHQ for each year from the current season back to the year the participant stared playing football. Finally, the third section of the PHQ is designed to produce information concerning engagement in other sport activities, including: (a) total sports played, and (b) total multi-sports hours. It contains a list of sports from which players were required to indicate those in which they have participated in regularly for at least a total minimum period of three months. Players were not required to record other sport activities engaged in during Physical Education (PE) classes in school. Total football and multi-sport hours were also included as a measure. The participants completed the PHQ in a classroom setting. Each participant was given one hour to complete the PHQ under supervision from the lead author, while allowing questions to facilitate individual understanding.

##### Six Measures from Postcode Data

Social classification and credit score are proxy indicators of socioeconomic status. In the UK, postcodes are associated with data pertaining to the locale to which they correspond. These data include income, employment, education, health, and crime levels, which can be accessed in multiple ways. For this study, the UK General Registrar Classification system was adopted that uses the average credit rating applying the Cameo™ geodemographic database. This provided a social classification (A, B, C1, C2, D, and E) determined by the UK’s Office for National Statistics and an average credit score (out of 999) for where each participant lives and goes to school. The social classification was scored numerically, with a higher score relating to a lower social classification (i.e., A = 1, B = 2, C1 = 3, C2 = 3, D = 4, and E = 5). The credit score denotes those with a higher score to have lower financial risk from ‘0’ (low) to ‘999’ (high). The participants area code was also included to test outline whether they are from urban and rural settings.

#### 2.1.3. Player Review Ratings

Player profiling is a widely used tool that is utilised within professional football academies [36]. Indeed, coach opinion is central to the subjective nature of youth football, with modern objective information readily available to professional coaches to support their judgement [37,38]. This study applied a unique progress assessment to measure each individual’s development. This tool, named the *43 Progression Steps*, applies a holistic approach during the player review process. This includes capturing the club’s pre-existing philosophy of developing core skills within *mental*, *physical*, *technical*, and *tactical* variables. These four sub-components grade specific characteristics that are considered necessary for development and progress towards senior professional status within this particular football club. The scoring system for the player profiling reports has a continual and progressive pattern rather than identical Likert scales. For example, the under-9 rating scale ranges from 1 (below average) to 4 (excellent), while the under-16 rating scale ranges from 26 (significantly below the required standard) to 33 (pushing towards the under-18 s). Throughout the development process, these specific grades are not prescribed within age groups, with players able to move through the tool seamlessly if they are developing or playing in certain areas above or below their chronological age.

The player review ratings were initially completed by the players who give their perception of themselves, and then the coaches subsequently provided their ratings alongside specific individual learning objectives. These reports were completed three times (i.e., pre-season, mid-season, and end of season), with each coach having completed the participants’ review ratings throughout the two seasons included in this study across the under-9 to under-16 age groups. Only the accumulated scores for all the components within each participant’s 43 Progression Steps rating were recorded at the start of season one and the end of season two in order to create two time points and analyse year-on-year developmental outcomes. Comparing the differences between the overall scores from the two player review ratings illustrated each player’s total development over two years, which was the score used for the data analysis in this current study. Two coaches from each age group (*n* = 16), who were deemed suitably qualified assessors (UEFA Pro, ‘A’, or ‘B’ Licenced alongside either the FA Advanced Youth Award or the FA Youth Award), graded each participant’s player review ratings for each of the specific characteristics. See Kelly’s doctoral thesis [34] for a comprehensive overview of the 43 Progression Steps player review tool.

#### 2.1.4. Data Analysis

The dataset was analysed via Lasso linear regression using cross-validated Lasso regression as implemented in the glmnet package in R [39]. Analysis of the improvement in player score across the two seasons used a coach assessed outcome measure, with the scores standardized at an age group aggregate:

$$y_{i,\;t}=\frac{x_{i,t}-\mu_{t}\;}{\sigma_{t}}$$

where $y_{i,t}$ is the corrected scores for the *i*th member of age group *t*, $x_{i,t}$ is the uncorrected scores for the *i*th member of age group *t*, $\mu_{t}$ is the mean of the $x_{i,t}$ scores and $\sigma_{t}$ is the standard deviation of the $x_{i,t}$ scores. The independent variables (IVs) were divided into categorical (“Home Postcode Social Grade”, “School Postcode Social Grade”, “PHV Status”, and “Birth Quarter”) and numeric (see SI for full list). Each numeric IV was standardized for mean at standard deviation at an age group aggregate, and the categorical IVs underwent a one-hot vector encoding [40]. Hence, coefficient estimates for numeric IVs reflect the change in DV per standard deviation from the average, while categorical IVs reflect the change in DV where the variable possess the relative value. The cross-validation technique first learned a model penalisation parameter, λ, by optimising the model performance characteristic (mean squared error) under 10-fold cross validation. The results for the optimal value of λ were then extracted to identify key contributing factors towards player review ratings.

### 2.2. Results

The summary of the Lasso regression techniques is outlined in Table 1. Results showed non-zero coefficients for improvement in subjective performance in 15 out of the 53 analysed features. Key findings revealed advanced percentage of predicted adult height (0.196), greater lob pass (0.160) and average dribble completion percentage (0.124), more total match-play hours (0.145), and an older relative age (BQ1 vs. BQ2: −0.133; BQ1 vs. BQ4: −0.060) were the most important features that contributed towards player review ratings.

### 2.3. Discussion

The purpose of this exploratory study was to examine the multidimensional factors that contributed to player review ratings across two seasons by applying a machine learning approach. Results showed a total of 15 of the 53 analysed features were important contributors towards player review ratings, which were representative of all four sub-components from the FCM (i.e., technical/tactical, physical, psychological, and social). Most notably, advanced percentage of predicted adult height, greater lob pass and average dribble completion percentage, more total match-play hours, and an older relative age were the largest features. Taken together, these findings underscore the holistic nature of the talent development processes in youth football.

Advanced percentage of predicted adult height had the greatest influence on player review ratings. The variation of maturation status (i.e., early, on-time, and late) between players within a single chronological age group can lead to up to 5-years difference in biological age [41]. The trainability and performance of physical competencies are closely aligned with maturity status [42]. Male players who experience their adolescent growth spurt mature earlier than their peers are invariably taller and heavier from late childhood and possess greater absolute and relative lean mass [43,44,45]. As a result of their advanced maturity, early maturing players also tend to outperform their less mature peers on tests of strength, power, speed, agility, and endurance [45,46]. However, from a psychological perspective, Cumming and colleagues [47] showed how later maturing players are more likely to possess and/or develop more adaptive self-regulation skills in the long-term, in particular self-evaluation and reflection. Moving forward, coaches should reflect on how an advanced maturity status can influence football-specific developmental outcomes from a holistic perspective (e.g., technical/tactical, physical, psychological, social). Since maturity status can significantly influence football-specific skills (e.g., physical competencies, self-regulation), coaches should observe and/or review players based on their maturity status (e.g., bio-banding [48]), rather than just their chronological age. This would support the long-term development of a wider pool of potential talent and focus on retaining later maturing players, whilst move the focus on short-term performance results that largely benefit early maturing players [25].

Greater lob pass and average dribble completion percentage (technical), as well as PCE ‘at’ and PCE ‘post’ (tactical), were important contributing features towards player review ratings. Coaches are the decision-makers in the player review rating process and often have a greater understanding of technical/tactical features compared to the other subcomponents of the FCM. Thus, it is not surprising that technical/tactical skills featured within these current results, as it is possible that greater value may be placed on these characteristics compared to the other subcomponents. This is emphasised by the traditional coach education and sport-specific qualifications that often focus on athlete competence compared to other developmental factors (e.g., confidence, connection, and character [6]) [49]. As such, although further evidence is needed, it is suggested coaches and organisations involve other stakeholders (e.g., Sport Scientists, Sport Psychologists, Strength and Conditioning Coaches) as part of a broader, holistic decision-making strategy when reviewing young players development [50].

There has been an ongoing search for the most appropriate activities that facilitate long-term player development in youth football (see Ford & Williams [51] for an overview). Findings from this current study found more total match-play hours had the largest contribution towards player review ratings. This may be explained through the coaches who are providing the players with their review rating being the same coaches who are selecting the players for the matches, and thus may be offering the players they perceive as better with more game time. However, it’s important to note that these coaches would have only been responsible for selecting these players in the recent years, whereas the *total* match-play hours accumulate the numbers the player has engaged in since they began playing football. Thus, another possible explanation is the benefits of engaging in match-play that may have contributed to player development. For instance, small-sided games have been shown to develop and refine young players’ skills and movements [52,53,54]. As such, coaches should consider how to offer a rich games programme to their players, through both competitive match-play and small-sided games, which could contribute to the holistic development of young players.

Birth quarter played an important role in influencing player review ratings, which favoured those born in the first three month of the year. This aligns with a wealth of relative age literature in youth soccer. As an example, initial research from Barnsley and colleagues [55] showed 45% of players selected for the 1989 U17 World Cup were born in the first three months of the annual selection year, whereas only 7.7% were born in the last three months of the annual selection year, with similar results shown across the U20 team squads. Since this preliminary research, the last three decades has generated various studies that shows how those born earlier in the selection year are overrepresented in talent pathways [56], accrue more league points [57], and win more games [58]. However, these benefits at youth level do not necessarily translate into success at adulthood in professional football [59,60,61]. As such, it is plausible to suggest that coaches perceive greater development in those who are relatively older largely due to their advanced age. As such, it is important for future research to explore the mechanisms of relative age effects and how they impact coaches perceived potential. Practitioners and researchers should also work collaboratively to design, implement, and evaluate a range of relative age solutions to help mitigate against these effects.

## 3. The Junior-to-Senior Transition from Youth Academy to Professional Level: Exploring the Characteristics of Selected and Deselected under-18 Players

Becoming a professional footballer is the aspiration of many academy prospects. However, it is well documented that only a small proportion of young players successfully graduate into senior professional levels. As an example, Dugdale and colleagues [62] showed how only 10% of 537 male players made the successful transition to professional level across a twelve-year period at a Scottish professional football club. Similarly, spanning an eleven-year period at an English professional football club, Kelly and colleagues [63] revealed how only 7.4% of 364 male players who entered the academy from under-9 to under-18 achieved a professional contract at aged 18 years. To better understand the junior-to-senior level transition, it is important to consider the characteristics that differentiate those academy players who achieve professional status and those who do not. By doing so, it will enable key stakeholders (e.g., coaches, practitioners, policy makers) employed in talent development programmes to allocate resources more efficiently, as well as facilitate a science-based support system [25].

The purpose of this study was to examine a range of factors based on the FCM (i.e., technical/tactical, physical, psychological, social) that may have contributed to under-18 academy football players achieving a professional contract.

### 3.1. Methods

#### 3.1.1. Sample

Eighteen under-18 male participants were recruited from the same tier four English professional football club and their category three academy. Their average weekly training and match-play time was 15 training hours/week and 1.5 match-play hours/week. Goalkeepers were not included in this study due to their contrasting position-specific requirements [26]. Parental consent and player assent were collected prior to the study commencing. The study was approved by the Ethics Committee of Sport and Health Sciences at the University of Exeter.

#### 3.1.2. Measures and Procedures

The same 53 factors from the eight measures outlined in Study 1 were collected for this study across two seasons (2014–2015 and 2015–2016). Player review ratings were also added since they were not used as an outcome measure. This dataset was then used to compare selected (i.e., offered a professional contract; *n* = 8) and deselected (i.e., not offered a professional contract; *n* = 10) players as they reached the end of their academy scholarship.

#### 3.1.3. Data Analysis

The dataset was analysed via Lasso regression techniques using cross-validated Lasso regression as implemented in the glmnet package in R. Analysis of the ‘Selection’ for professional play was performed using binomial Lasso regression, coding the outcome as 1 for ‘Selected‘ and 0 for ‘Deselected’. The cross-validation technique first learned a model penalisation parameter, λ, by optimising the model performance characteristic (binomial deviance) under 10-fold cross validation. The results for the optimal value of λ were then extracted to identify key contributing factors. In reporting the results of the binomial Lasso regression, the exponential of the coefficients was included. In the case of a logistic binomial mode, the exponential of the coefficients is equivalent to the change in odds ratio for each increase of the dependent variable by 1, one standard deviation in this case [13,40].

### 3.2. Results

The summary of the Lasso regression techniques are outlined in Table 2. The relatively small parameter space of importance in Table 2 is not indicative that few features matter, but instead due to the limited quantity of data available. The size of the effect of the psychological factor, while arising from a limited quantity of data, should be noted. Having included the possibility for multiple confounding factors, the strongest marker for signing was the psychological outcomes of the player. What is not clear, given the observational nature of the study, is if improvements in psychological factors would lead to a greater chance of signing a professional contract or if it is in fact a proxy variable marking out players with a range of sought after factors.

The prominence of Factor 3 (coping with performance and developmental pressures) with regard to both end points posed a key question: is this factor more prominent than the other five factors, or are all the six closely correlated in the dataset and the Lasso is selecting the most informative? To quantify the relative associations of the six PCDEQ factors, the correlation matrix was calculated for the progression data set. Of the PCDEQ factors, Factor 3 only shows a reasonably strong correlation with Factor 4 (ability to organise and engage in quality practice), with only weak links to the other terms. Hence, we conclude that PCDEQ Factor 3 and Factor 4 are the strongest discriminatory variables relating to signing a professional contract. Hence, it appears valid to suggest Factors 1, 2, 5, and 6 may pose no contribution to signing a professional contract.

### 3.3. Discussion

The junior-to-senior transition is arguably the most defining moment in a promising young player’s career. Indeed, by achieving their first professional contract, a player moves one-step closer to fulfilling their aspirations of competing for their respective senior first team. To the author’s knowledge, this was the first study to explore the characteristics of selected and deselected under-18 academy players using machine learning techniques. Key findings revealed how PCDEQ Factor 3 and Factor 4 were important contributing factors towards achieving a professional contract. Moreover, player review ratings (i.e., higher coach scores), slalom dribble (i.e., quicker dribble times), and a lower home social classification (i.e., derived from more deprived areas) also provided a small contribution.

PCDEQ Factor 3 and Factor 4 were important contributing factors within this current study. Indeed, psychological factors have been previously identified as important attributes that are required during the junior-to-senior transition. As an example, the current findings are consistent with previous studies that found ‘good developers’ within team sports had a significantly greater perceived ability to cope with performance and developmental pressures (e.g., such as overcoming struggles, set-backs, injury, or a decline in performance) compared to ‘poor developers’ [32]. These current findings also compliment the opinions of coaches as derived from qualitative studies. First, Mills and colleagues’ [64] analysis of ten expert coaches revealed six factors, including resilience, that were perceived to either positively or negatively influence player development. Second, Cook and colleagues [65] reported four general dimensions of mental toughness, including competitiveness with self and others, mind-set, resilience, and personal responsibility, that are associated with the ability to cope with the pressures inherent in the academy environment. Similarly, Holt and Mitchell [66] identified a deficiency in coping behaviours of professional football players near to being released, whereas Holt and Dunn [67] revealed how discipline, commitment, resilience, and social support were associated with becoming a professional football player. While it is plausible to suggest that these psychological characteristics are generally accepted as crucial factors for positive developmental outcomes, further investigation is required to design, implement, and evaluate effective psychological development strategies within academy environments [68]

When compared to other specialist support staff in youth soccer environments (i.e., Sport Scientists, Strength and Conditioning Coaches, Performance Analysts), Sport Psychologists appear to be less common (particularly in a full-time capacity) [68]. Since the development of psychological characteristics appears to be an important contributing factor towards both coaches perceived development outcomes (i.e., Study 1) as well as achieving a professional contract (i.e., Study 2), professional clubs and governing bodies should consider how they can formalise their psychological support and invest in qualified practitioners. By doing so, it will enable young players the opportunity to access psychological support when required, as well as help with coach development to ensure effective strategies are consistently implemented throughout coaching provision [69].

Unsurprisingly, player review ratings contributed to selection. This is likely due to the fact that those coaches who are rating the players are the same stakeholders who are part of the professional contract decisions. More unexpectedly, dribbling ability and social classification also made a small contribution towards selection. First, dribbling has been previously identified as an important technical attribute as part of a multidisciplinary study when comparing selected and deselected players [2]. Thus, these current findings further support the significance of possessing ball dribbling skills, which could be incorporated into developmental programmes to ensure players are adequately prepared as they navigate their ways towards senior levels. Second, the results of a lower home social classification contributing to achieving a professional contract reflect the stereotype of football being a sport participated by individuals with a lower socioeconomic status [70]. This might imply that football retains a traditional divide between socioeconomic status and participation [71], which may have implications on opportunities to achieve a professional contract [72]. However, it is important to consider the exploratory nature of these findings, as well as the limited number of participants included in this current study. Therefore, future research is encouraged to further explore the significance of possessing ball dribbling skills and the role of socioeconomic status in developing expertise in football.

## 4. Limitations

The key limitations of this study are the role of retrospective analysis and predictive models. The techniques used here are best viewed as exploratory and hypothesis generating, rather than confirmatory, as they do not seek to provide evidence for or against any pre-existing mechanisms but generate new insight and an optimal predictive model given the available data. In the case of the ‘Selection’ analysis, the inherently small dataset does provide limitations to the analysis, whereby machine learning approaches with small datasets inherently run the risk of memorising the sample rather than generating transferable lessons [73]. In general, the subset selection algorithms demonstrated here could provide stakeholders within the football development community with insight into the operational data currently held. Data collection and storage mechanisms have increasingly become cheaper over the past two decades (e.g., the rise of wearable technology and cloud storage). Moving forward, an important question is how to leverage such data to aid decision making. Techniques such as those shown here are invaluable in being able to quickly and easily reduce data to interpretable models and highlight key signals.

## 5. Conclusions

It appears the key factors associated with positive developmental outcomes in youth soccer are not always technical and tactical in nature, which is where youth coaches often have their expertise and/or focus their attention on talent development. Indeed, the relative importance of these factors is likely to change over time, and with age, although psychological attributes appear to be influential to reaching potential across the academy journey. Therefore, coaches are encouraged to focus on long-term potential as opposed to short-term performance. The techniques used here also serves as an impetus for researchers to adopt machine learning approaches when analysing multidimensional databases for talent development purposes.

## Figures and Tables

**Table 1:** Summary of non-zero coefficients for improvement in subjective performance.

Feature	Coefficient/SD of Feature
Ball juggling	0.083
Lob pass	0.160
Average dribble competition percentage	0.124
PCE ‘at’	0.091
PCE ‘post’	0.062
PCDEQ Factor 3	0.062
Total match-play hours	0.145
Total individual practice hours	−0.027
0–30 m sprint	−0.041
CMJ height	0.053
Percentage of predicted adult height attained	0.196
Birth quarter 2 (reduced relative to birth quarter 1)	−0.133
Birth quarter 4 (reduced relative to birth quarter 1)	−0.060
Home postcode social grade 2 (reduced in comparison to social grade 1 or 4)	−0.082
School postcode social grade 3 (reduced relative to social grade 1 or 4)	−0.045

**Table 2:** Summary of non-zero coefficients for likelihood of signing a professional contract.

Feature	Coefficient/SD of Feature	Odds Ratio/SD of Feature
43 progression steps rating	0.64	1.89
Slalom dribble	0.01	1.01
PCDEQ Factor 3	0.44	1.55
Home postcode social grade 2	−0.12	0.89

## Data Availability

Data can be obtained via the lead author.

## References

[1] Sarmento H., Anguera M.T., Pereira A., Araújo D. (2018). Talent Identification and Development in Male Football: A Systematic Review. Sports Med..

[2] Huijgen B.C.H., Elferink-Gemser M.T., Lemmink K.A.P.M., Visscher C. (2014). Multidimensional performance char-acteristics in selected and deselected talented soccer players. Eur. J. Sport Sci..

[3] Forsman H., Gråstén A., Blomqvist M., Davids K., Liukkonen J., Konttinen N. (2016). Development of perceived competence, tactical skills, motivation, technical skills, and speed and agility in young soccer players. J. Sports Sci..

[4] Zuber C., Zibung M., Conzelmann A. (2016). Holistic patterns as an instrument for predicting the performance of prom-ising young soccer players—A 3-years longitudinal study. Front. Psychol..

[5] Côté J., Murata A., Turnnidge J., Hancock D.J., Kelly A., Côté J., Jeffreys M., Turnnidge J. (2021). Situating birth advantages within the youth sport system. Birth Advantages and Relative Age Effects in Sport: Exploring Organizational Structures and Creating Appropriate Settings 2.

[6] Côté J., Turnnidge J., Evans M.B. (2014). The dynamic process of development through sport. Kinesiol. Slov. Sci.-Entific J. Sport.

[7] Kelly A.L., Wilson M.R., Williams C.A. (2018). Developing a football-specific talent identification and development profiling concept—The Locking Wheel Nut Model. Appl. Coach. Res. J..

[8] The Football Association (2020). The FA’s 4 Corner Model [Online]. https://thebootroom.thefa.com/resources/coaching/the-fas-4-corner-model.

[9] Towlson C., Cope E., Perry J.L., Court D., Levett N. (2019). Practitioners’ multi-disciplinary perspectives of soccer talent according to phase of development and playing position. Int. J. Sports Sci. Coach..

[10] Kelly A.L., Wilson M.R., Jackson D.T., Goldman D.E., Turnnidge J., Côté J., Williams C.A. (2021). A multidisciplinary investigation into “playing-up” a chronological age group in an English football academy. J. Sports Sci..

[11] Goldman D.E., Turnnidge J., Côté J., Kelly A.L., Kelly A.L., Côté J., Jeffreys M., Turnnidge J. (2021). “Playing-up” in youth soccer. Birth Advantages and Relative Age Effects in Sport: Exploring Organizational Structures and Creating Appropriate Settings.

[12] Turnnidge J., Kelly A.L., Kelly A., Côté J., Jeffreys M., Turnnidge J. (2021). Organizational structures: Looking back and looking ahead. Birth Advantages and Relative Age Effects in Sport: Exploring Organizational Structures and Creating Appropriate Settings.

[13] Tibshirani R. (1996). Regression shrinkage and selection via the Lasso. J. R. Stat. Soc. Ser. B (Method-Ological).

[14] Oquendo M.A., Baca-García E., Artés A., PerezCruz F., Galfalvy H., Blascofontecilla H., Madigan D., Duan N. (2012). Machine learning and data mining: Strategies for hypothesis generation. Mol. Psychiatry.

[15] Bunker R., Thabtah F. (2019). A machine learning framework for sport result prediction. Appl. Comput. Inform..

[16] Unni M.P., Menon P.P., Livi L., Wilson M.R., Young W.R., Bronte-Stewart H.M., Tsaneva-Atanasova K. (2020). Data-Driven prediction of freezing of gait events from stepping data. Front. Med. Technol..

[17] Barth M., Emrich E., Güllich A. (2019). A machine learning approach to “revisit” specialization and sampling in institu-tionalized practice. SAGE Open.

[18] Jauhiainen S., Kauppi J.-P., Leppänen M., Pasanen K., Parkkari J., Vasankari T., Kannus P., Äyrämö S. (2020). New machine learning approach for detection of injury risk factors in young team sport athletes. Laryngo-Rhino-Otologie.

[19] Jones B.D., Hardy L., Lawrence G., Kuncheva L.I., Brandon R., Bobat M., Thorpe G. (2020). It ain’t what you do—It’s the way that you do it: Is optimizing challenge key in the development of super-elite batsmen?. J. Expert..

[20] Jones B.D., Hardy L., Lawrence G., Kuncheva L.I., Du Preez T., Brandon R., Such P., Bobat M. (2019). The identification of “game changers” in England cricket’s developmental pathway for elite spin bowling: A machine learning approach. J. Expert..

[21] Musa R.M., Majeed A.P.P.A., Taha Z., Chang S.W., Nasir A.F.A., Abdullah M.R. (2019). A machine learning ap-proach of predicting high potential archers by means of physical fitness indicators. PLoS ONE.

[22] Owen J., Owen R., Hughes J., Leach J., Anderson D., Jones E. (2022). Psychosocial and physiological factors affecting selection to regional age-grade rugby union squads: A machine learning approach. Sports.

[23] Musa R.M., Majeed A.P.P.A., Konsi N.A., Abdullah M.R. (2020). Machine Learning in Team Sports: Performance Analysis and Talent Identification in Beach Soccer & Sepak-Takraw.

[24] The Premier League (2011). Elite Player Performance Plan [Online]. https://www.premierleague.com/youth/EPPP.

[25] Kelly A.L., Williams C.A. (2020). Physical characteristics and the talent identification and development processes in male youth soccer: A narrative review. Strength Cond. J..

[26] Gil S.M., Zabala-Lili J., Bidaurrazaga-Letona I., Aduna B., Lekue A.B., Santos-Concejero J., Granados C. (2014). Talent identification and selection process of outfield players and goalkeepers in a professional soccer club. J. Sports Sci..

[27] Vaeyens R., Malina R.M., Janssens M., Van Reterghem B., Bourgois J., Vrijens J., Philippaerts R.M. (2006). A multidis-ciplinary selection model for youth soccer: The Ghent Youth Soccer Project. Br. J. Sports Med..

[28] Kelly A., Wilson M.R., Jackson D.T., Williams C.A. (2020). Technical testing and match analysis statistics as part of the talent development process in an English football academy. Int. J. Perform. Anal. Sport.

[29] Kelly A.L., Wilson M.R., Jackson D.T., Turnnidge J., Williams C.A. (2020). Speed of thought and speed of feet: Examining perceptual-cognitive expertise and physical performance in an english football academy. J. Sci. Sport Exerc..

[30] Kelly A.L., Williams C.A., Jackson D.T., Turnnidge J., Reeves M.J., Dugdale J.H., Wilson M.R. Considering the role of socioeconomic status and psychological characteristics in talent development: An exploratory study in an English soccer academy. Sci. Med. Footb. submitted.

[31] MacNamara A., Collins D. (2011). Development and initial validation of the Psychological Characteristics of Developing Excellence Questionnaire. J. Sport Sci..

[32] MacNamara A., Collins D. (2013). Do mental skills make champions? Examining the discriminant function of the psy-chological characteristics of developing excellence questionnaire. J. Sport Sci..

[33] Ford P., Ward P., Hodges N.J., Williams A.M. (2009). The role of deliberate practice and play in career progression in sport: The early engagement hypothesis. High Abil. Stud..

[34] Kelly A.L. (2018). A Multidisciplinary Investigation into the Talent Identification and Development Process in an English Football Academy. Ph.D. Thesis.

[35] Khamis H.J., Roche A.F. (1994). Predicting adult stature without using skeletal age: The Khamis-Roche method. Pediatrics.

[36] Morley D., Morgan G., McKenna J., Nicholls A.R. (2014). Developmental contexts and features of elite academy football players: Coach and player perspectives. Int. J. Sports Sci. Coach..

[37] Sieghartsleitner R., Zuber C., Zibung M., Conzelmann A. (2019). science or coaches’ eye?—Both! beneficial collaboration of multidimensional measurements and coach assessments for efficient talent selection in elite youth football. J. Sports Sci. Med..

[38] Tangalos C., Robertson S.J., Spittle M., Gastin P.B. (2015). Predictors of individual player match performance in junior australian football. Int. J. Sports Physiol. Perform..

[39] Friedman J.H., Hastie T., Tibshirani R. (2010). Regularization paths for generalized linear models via coordinate descent. J. Stat. Softw..

[40] Dunn P.K., Smyth G.K. (2018). Generalized Linear Models with Examples in R.

[41] Malina R.M., Bouchard C., Bar-Or O. (2004). Growth, Maturation and Physical Activity.

[42] Lloyd R.S., Oliver J.L. (2012). The Youth Physical Development Model: A new approach to long-term athletic development. Strength Cond. J..

[43] Clemente F.M., Clark C.C.T., Leão C., Silva A.F., Lima R., Sarmento H., Figueiredo A.J., Rosemann T., Knechtle B. (2021). Exploring relationships between anthropometry, body composition, maturation, and selection for competition: A study in youth soccer players. Front. Physiol..

[44] Malina R.M., Eisenmann J.C., Cumming S.P., Ribeiro B., Aroso J. (2004). Maturity-associated variation in the growth and functional capacities of youth football (soccer) players 13–15 years. Eur. J. Appl. Physiol..

[45] Meylan C., Cronin J., Oliver J., Hughes M. (2010). Talent identification in soccer: The role of maturity status on physical, physiological and technical characteristics. Int. J. Sports Sci. Coach..

[46] Guimarães E., Ramos A., Janeira M.A., Baxter-Jones A.D.G., Maia J. (2019). How does biological maturation and training experience impact the physical and technical performance of 11–14-year-old male basketball players?. Sports.

[47] Cumming S.P., Searle C., Hemsley J.K., Haswell F., Edwards H., Ryan D., Scott S., Gross A., Lewis J., White P. (2018). Biological maturation, relative age and self-regulation in male professional academy soccer players: A test of the underdog hypothesis. Psychol. Sport Exerc..

[48] Cumming S.P., Lloyd R.S., Oliver J.L., Eisenmann J.C., Malina R.M. (2017). Bio-banding in sport: Applications to competition, talent identification, and strength and conditioning of youth athletes. Strength Cond. J..

[49] Lefebvre J.S., Evans M.B., Turnnidge J., Gainforth H.L., Côté J. (2016). Describing and classifying coach development programmes: A synthesis of empirical research and applied practice. Int. J. Sports Sci. Coach..

[50] Piggott B., Müller S., Chivers P., Papaluca C., Hoyne G. (2019). Is sports science answering the call for interdisciplinary research? A systematic review. Eur. J. Sport Sci..

[51] Ford P.R., Williams M.A., Baker J., Cobley S., Schorer J., Wattie N. (2017). Sport Activity in Childhood: Early Specialization and Diversification. Routledge Handbook of Talent Identification and Development in Sport.

[52] Coutinho D., Gonçalves B., Santos S., Travassos B., Wong D.P., Sampaio J. (2019). Effects of the pitch configuration design on players’ physical performance and movement behaviour during soccer small-sided games. Res. Sport. Med..

[53] Santos S., Coutinho D., Gonçalves B., Schöllhorn W., Sampaio J., Leite N. (2018). Differential Learning as a Key Training Approach to Improve Creative and Tactical Behavior in Soccer. Res. Q. Exerc. Sport.

[54] Travassos B., Duarte R., Vilar L., Davids K., Araújo D. (2012). Practice task design in team sports: Representativeness enhanced by increasing opportunities for action. J. Sport. Sci..

[55] Barnsley R.H., Thompson A.H., Legault P. (1992). Family planning: Football style. Relat. Age Eff. Football. Int. Rev. Sociol. Sport.

[56] Finnegan L., Richardson D., Littlewood M., McArdle J. (2017). The influence of date and place of birth on youth player selection to a National Football Association elite development programme. Sci. Med. Footb..

[57] Verbeek J., Lawrence S., van der Breggen J., Kelly A.L., Jonker L., Kelly A., Côté J., Jeffreys M., Turnnidge J. (2021). The average team age method and its potential to reduce relative age effects. Birth Advantages and Relative Age Effects in Sport: Exploring Organizational Structures and Creating Appropriate Settings.

[58] Söderström T., Brusvik P., Lund S. (2019). Factors underlying competitive success in youth football. A study of the Swedish national U15 football talent system. Scand. Sport Stud. Forum.

[59] Ashworth J., Heyndels B. (2007). Selection bias and peer effects in team sports: The effect of age grouping on earnings of German soccer players. J. Sports Econ..

[60] Grossmann B., Lames M. (2013). Relative Age Effect (RAE) in Football Talents–the Role of Youth Academies in Transition to Professional Status in Germany. Int. J. Perform. Anal. Sport.

[61] Ramos-Filho L., Ferreira M.P. (2021). The reverse relative age effect in professional soccer: An analysis of the Brazilian National League of 2015. Eur. Sport Manag. Q..

[62] Dugdale J.H., Sanders D., Myers T., Williams A.M., Hunter A.M. (2021). Progression from youth to professional soccer: A longitudinal study of successful and unsuccessful academy graduates. Scand. J. Med. Sci. Sport.

[63] Kelly A.L., Wilson M.R., Gough L.A., Knapman H., Morgan P., Cole M., Jackson D.T., Williams C.A. (2020). A longitudinal investigation into the relative age effect in an English professional football club: The ‘underdog hypothesis’. Sci. Med. Footb..

[64] Mills A., Butt J., Maynard I., Harwood C. (2012). Identifying factors perceived to influence the development of elite youth football academy players. J. Sport Sci..

[65] Cook C., Crust L., Littlewood M., Nesti M., Allen-Collinson J. (2014). ‘What it takes’: Perceptions of mental toughness and its development in an English Premier League Soccer Academy. Qual. Res. Sport Exerc. Health.

[66] Holt N.L., Mitchell T. (2006). Talent development in English professional football. Int. J. Sport Psychol..

[67] Holt N.L., Dunn J.G.H. (2004). Towards a grounded theory of the psychosocial competencies and environmental condi-tions associated with soccer success. J. Appl. Sport Psychol..

[68] Pain M.A., Harwood C.G. (2004). Knowledge and perceptions of sport psychology within English soccer. J. Sport Sci..

[69] Harwood C., Barker J.B., Anderson R.J. (2015). Psychosocial development in youth soccer players: Assessing the effec-tiveness of the 5C’s intervention program. Sport Psychol..

[70] Bourke A. (2003). The dream of becoming a professional soccer player. J. Sport Soc. Issues.

[71] Winn C.O.N., Ford P.R., McNarry M.A., Lewis J., Stratton G. (2016). The effect of deprivation on the developmental activities of adolescent rugby union players in Wales. J. Sport Sci..

[72] Lawrence D. (2017). Sociodemographic profile of an Olympic team. Public Health.

[73] Zhang Y., Ling C. (2018). A strategy to apply machine learning to small datasets in materials science. NPJ Comput. Mater..

